# Evaluating the Clinical Risk Index for Babies (CRIB) II Score for Mortality Prediction in Preterm Newborns: A Prospective Observational Study at a Tertiary Care Hospital

**DOI:** 10.7759/cureus.58672

**Published:** 2024-04-21

**Authors:** Heena Bhandekar, Swapnali Bansode Bangartale, Ishani Arora

**Affiliations:** 1 Paediatrics, Datta Meghe Medical College, Datta Meghe Institute of Higher Education & Research, Nagpur, IND; 2 Paediatrics, Narendra Kumar Prasadrao (NKP) Salve Institute of Medical Sciences & Research Centre and Lata Mangeshkar Hospital, Nagpur, IND

**Keywords:** mortality predictor, sick neonate, neonatal mortality, clinical risk index for babies score, preterm newborns

## Abstract

Introduction: Neonatal mortality is an issue that affects both the developed and developing world. It is very important in the neonatal intensive care unit (NICU) to do the assessment of the severity of neonatal illness, which in turn helps in estimating and preventing mortality in the NICU by improving healthcare control and by rational use of resources. This research was carried out to evaluate how effectively the Clinical Risk Index for Babies (CRIB) II score can predict mortality rates among newborns treated in our NICU.

Methodology: This prospective observational study spanned one year, commencing in October 2021 and concluding in September 2022, within the confines of our NICU. The CRIB II score calculation was performed for included newborns, and the outcomes of the newborns were compared. A receiver operating characteristic (ROC) curve was obtained to ascertain the optimal CRIB II cut-off score for predicting mortality.

Results: Within the designated research timeframe, 292 neonates were admitted to the NICU. Forty-four newborns were enrolled in the study. Preterm neonates who died had higher CRIB II scores than those who survived, and their median (IQR) was 6 (1-12) vs. 9.5 (5-14) (p=0.0003). The estimate for the area under the curve was 0.83 (95% CI 0.68-0.92), and the odds ratio of 2.56 suggests neonates with a higher CRIB II score have higher chances of mortality.

Conclusion: The CRIB II score is very good at predicting mortality in preterm newborns.

## Introduction

Neonatal mortality is a global issue. As per the National Family Health Survey 5 (NFHS-5) conducted from 2019 to 2021, there are 24.9 newborn deaths for every 1000 live births in India [1]. The commonest causes of mortality in neonates are preterm birth, birth asphyxia, and sepsis. Preterm births are defined as births that occur before the full 37 weeks of pregnancy [2]. It is very important in the neonatal intensive care unit (NICU) to evaluate the severity of neonatal illness which, in turn, helps in estimating and preventing in-hospital mortality in NICU by improving healthcare control and rational use of resources [3]. Therefore, the risk prediction rules and scoring systems are gaining importance with time to measure the severity of the clinical parameters of the patient and do stratification of patients accordingly [4]. The most widely described severity scores for neonatal mortality and morbidity in the literature are as follows: Clinical Risk Index for Babies (CRIB), CRIB II, Score for Neonatal Acute Physiology (SNAP), SNAP II, Score for Neonatal Acute Physiology with Perinatal Extension (SNAP-PE), and SNAP-PE II [5,6].

The International Neonatal Network in the United Kingdom first developed the CRIB score in 1993. In this score, data gathered during the first 12 hours after birth is used for evaluating the morbidity and mortality rates in hospitals [7]. In 2003, CRIB was updated to the CRIB II score by eliminating potential problems that may be caused by the use of medicines and human error caused during it, making it a simplified scoring system [8]. The CRIB score consists of six variables, i.e., congenital malformations, birth weight, gestational age, maximal base excess checked within the initial 12 hours of admission to the hospital, and minimum and maximum oxygen consumption. However, CRIB II is composed of five variables, including birth weight, sex of the baby, body temperature on admission, maximum base excess, and gestational age. With this background, the study aimed to assess the efficiency of the CRIB II score in the prediction of mortality risk in preterm neonates admitted to the NICU.

## Materials and methods

During the specified period from October 2021 to September 2022, our prospective observational study was carried out at the NICU of Narendra Kumar Prasadrao Salve Institute of Medical Sciences & Research Centre (NKPSIMS & RC) and Lata Mangeshkar Hospital (LMH), Nagpur, India. The primary objective was to gain valuable insights into the health outcomes and care practices for newborns within this critical medical setting. Approval from the Institutional Ethics Committee of NKPSIMS & RC and LMH (approval number: 11/2021) was obtained before commencing the study. To ensure ethical conduct, informed written consent was recorded from the parents of the newborns, signifying their agreement to have their baby participate in the study. Newborns, whose parents were not willing to participate, were excluded.

The enrollment process involved consecutively enlisting participants, ensuring a representative sample of newborns admitted to the NICU during the study timeframe. This approach aimed to obtain a diverse range of cases, allowing better analysis of various factors influencing neonatal health and care.

Our study included all preterm newborns having gestational age of <32 weeks, who needed admission to the NICU during the initial 12 hours of life. The babies excluded from the study were those newborns with birth weight <500 g, those who needed admission to the NICU after 12 hours of life, and those who expired during the first 12 hours of life, as well as those babies who had lethal congenital malformations or those who were discharged against medical advice.

One crucial aspect of our investigation was calculating the CRIB II score. This scoring system was applied after a thorough evaluation of each newborn within the first 12 hours of admission, after the stabilization of their medical condition. This is a well-established method in neonatal medicine, designed to assess the clinical risk and predict outcomes for critically ill newborns. By utilizing this scoring mechanism, we sought to contribute valuable information to the existing body of knowledge regarding the prognostic indicators for infants in the NICU setting. The birth weight was recorded for each baby on admission to the NICU on the electronic weighing machine (Phoenix electronic weighing scale, India) (±5 g error) without clothing. Gestational age was determined using the modified Ballard score within the first 24 hours of birth [9]. Body temperature was measured on admission after initial stabilization using a digital thermometer. All babies were screened for the presence of any congenital abnormalities. Oxygen saturation was checked in the NICU on a Schiller Truscope 2 multipara monitor, in Switzerland. Blood gas was recorded using a Siemens fully automatic arterial blood gas (ABG) machine (Germany) available in our NICU at birth and then as needed according to the clinical condition of each infant. The reading of the maximum base excess checked in the initial 12 hours was recorded.

According to the CRIB II scoring system, each of the following five parameters, i.e., sex of the baby, birth weight, temperature, maximum base excess, and gestational age, was recorded. The CRIB II score was then obtained by adding individual scores of these parameters. Neonatal mortality reflects death before discharge from the NICU.

The data was entered in MS Excel and then coded as needed. Stata Statistical Software: Release 14 (2015; StataCorp LLC, College Station, Texas, United States) was used for the analysis. Survival outcomes were subjected to comparative analysis utilizing the Mann-Whitney test to discern potential distinctions between the groups of survivors and non-survivors. An assessment of the predictive strength of the CRIB II score for neonatal mortality was conducted through the utilization of a receiver operating characteristic (ROC) curve. The p-value is statistically significant at 0.05 or less.

## Results

During our study period, there were 292 admissions to the NICU. Among these, eight neonates died in 12 hours, 43 were discharged against medical advice, and 20 had severe congenital malformations. Furthermore, 30 neonates did not have complete data or whose parents did not consent to study participation. One hundred twenty neonates were excluded because they were hemodynamically stable at admission and ABG was not done in them. Of the remaining, 35 babies had gestational age greater than or equal to 32 weeks. Therefore, 44 preterm newborns qualified and were enrolled in this study. They were followed up to discharge from NICU or their death.

Table 1 and Table 2 provide the demographic and other characteristics of the study group. The median age of the newborns was one hour at admission. The study population included 25 males (56.82%) and 19 females (43.18%). The median gestational age of the newborns, who were enrolled in the study, was 31 weeks, and the range was from 26 weeks to 32 weeks. The median stay duration for preterm newborns was 10.5 days (range of 1-65 days). Twenty-eight (63.64%) inborn babies were included, while 16 (36.36%) babies born outside and referred to our center were included in the study. The median weight of neonates was 1160 grams, while the range was 720-1800 grams. The median Apgar score at five minutes was 8, while the range was 5-9. Of the study population, 18 newborns survived (40.91%), while 26 babies died (59.09%).

**Table 1:** Demographic characteristics of the study population

Variables		Median (percentage)
Gender	Female	19 (43.18%)
	Male	25 (56.82%)
Inborn		28 (63.64%)
Outborn		16 (36.36%)
Outcome	Survivors	18 (40.91%)
	Deaths	26 (59.09%)

**Table 2:** Other characteristics of the study population

Variables	Median (range)
Age in hours on admission	1 (1-13)
Gestational age (weeks)	31 (26-32)
Duration of hospital stay	10.5 (1-65)
Birth weight (gram)	1160 (720-1800)
Apgar score at five minutes	8 (5-9)

Table 3 shows an association between different factors with the outcome of newborns. The median age at which survivors were admitted in this study was one hour with a range of 1-13 hours, the same as that of infants who have died, that is, one hour with a range of 1-12 hours. The odds ratio of survival for each hour increase in age at admission was 0.87, indicating lower odds of survival for each hour delay at admission, and this association is statistically insignificant (p=0.3272). The median gestation age of survivors was 31 weeks, while that of babies who died was 29 weeks, with a range of 26-32 weeks in both groups. The odds ratio of survival for each week increase in gestational age was 0.64, indicating lower odds of survival with increasing gestational age, but this association is statistically not significant (p=0.1902). The median duration of hospital stay for survivors was 23.5 days with a range of 6-65 days, while that for babies who died was six days (range of 1-22 days). The odds of survival for an increase in the duration of hospital stay on each day was 0.67, indicating lower odds of survival with increasing stay duration, and this association is statistically significant (p<0.0001). The median birth weight of survivors is 1310 grams with a range of 920-1800 grams, while that of babies who died was 1100 grams with a range of 720-1550 grams. The odds of survival for each gram of increase in birth weight were 0.99, suggesting that there was no association between the survival of neonates and birth weight; however, this association is statistically significant (p=0.0064). 

**Table 3:** Association of different factors and outcome NS: not significant; HS: highly significant

Factors	Survivors		Deaths		Odds ratio (95% confidence interval)	P-value
	Median	Range	Median	Range		
Age in hours	1	1-13	1	1-12	0.87 (0.70-1.08)	0.3272, NS
Gestational age (weeks)	31	26-32	29	26-32	0.64 (0.41-1.00)	0.1902, NS
Duration of stay	23.5	6-65	6	1-22	0.67 (0.51-0.87)	<0.0001, HS
Birth weight (gram)	1310	920-1800	1100	720-1550	0.99 (0.990-0.997)	0.0064, HS
Apgar score at five minutes	8	5-9	8	6-9	0.90 (0.51-1.59)	0.9406, NS

The median Apgar score in both survivors and babies who died is 8, with a range of 5-9 in survivors and 6-9 in babies who died. The odds of survival for each point increase in the Apgar score is 0.90, suggesting that there is hardly any association between the Apgar score and the survival of babies and this association is not statistically significant (0.9406). Of the 18 newborns who survived, 11 (61.11%) were males, while seven (38.89%) were females. Of the 26 newborns who died, 14 were males (53.84%), while 12 were females (46.15%). The odds of survival for male infants compared to female infants was 0.46, suggesting lower odds of survival chances in males, but this association is not statistically significant (p=0.632). Of the 18 survivors, 10 were inborn (55.56%) and eight (44.44%) were outborn, and among babies who died, 18 (69.23%) were inborn and 8 (30.77%) were outborn. The odds of survival for inborn babies were higher than for outborn babies since the odds ratio was 2.25, but this association is statistically insignificant (p=0.354) (Table 4).

**Table 4:** Association of demographic characteristics and outcome NS: not significant

Factors	Survivors		Deaths		Odds ratio (95% confidence interval)	P-value
	Median	Percentage	Median	Percentage		
Male	11	61.11%	14	53.84%	0.46 (0.08-2.22)	0.632, NS
Female	7	38.89%	12	46.15%		
Inborn	10	55.56%	18	69.23%	2.25 (0.48-10.46)	0.354, NS
Outborn	8	44.44%	8	30.77%		

**Figure 1:**
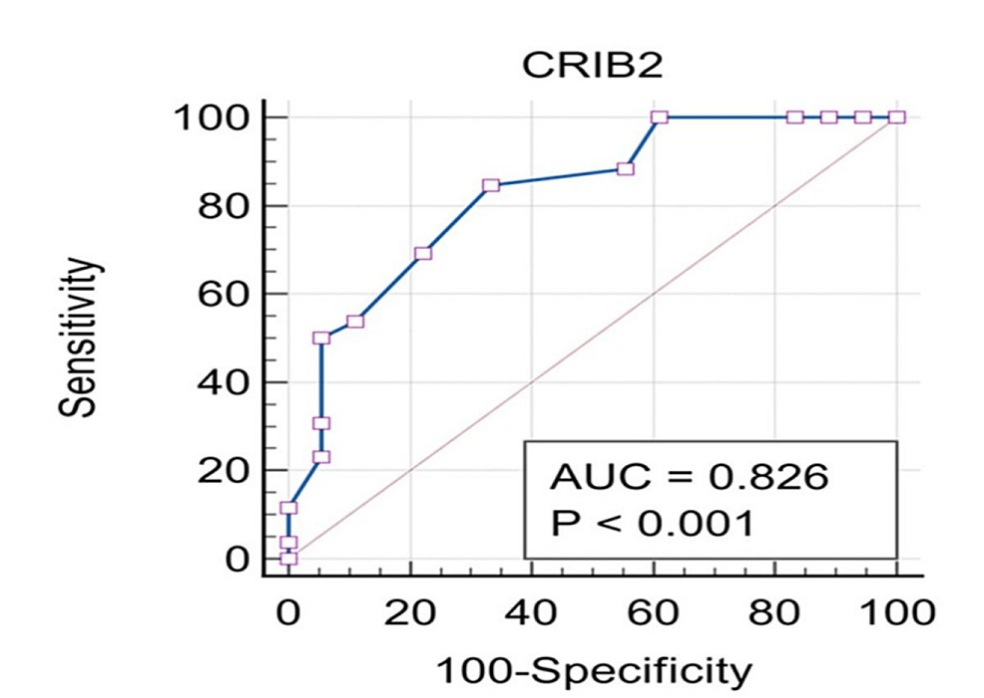
Receiver operating characteristics curve CRIB II: Clinical Risk Index for Babies II; AUC: area under the curve

The median CRIB II score among survivors was 6 with a range of 1-12, while among those who died, it was 9.5 with a range of 5-14. In this study, the estimate of the area under the curve (AUC) was 0.826, and the confidence interval ranged from 0.68 to 0.92 (Figure 1). The specificity and sensitivity of the CRIB II score in forecasting mortality among preterm neonates were determined as 84.62% and 66.67%, respectively. The p-value is <0.0001, which is highly significant statistically. The cut-off value of the present study is >7, which suggests a CRIB II score of >7 was significantly associated with neonatal mortality (Table 5).

**Table 5:** Area under the curve for ROC AUC: area under the curve; HS: highly significant; ROC: receiver operating characteristic

AUC	95% confidence interval	Sensitivity	Specificity	Z-value	P-value	Best cut-off
0.83	0.68-0.92	84.62	66.67	5.075	<0.0001, HS	>7

The neonates are divided into two groups according to the CRIB II score of >7 or ≤7 in Table 6, which gives an association between mortality in neonates and the CRIB II score. Of the 26 babies who died, 18 neonates had a CRIB II score of >7, and 8 had a score of ≤7. Four of the 18 surviving newborns had a CRIB II score of >7, while 14 newborns had a score of ≤7. The odds ratio of 7.87 suggests increased odds of mortality chances with increasing CRIB II score, and this association is statistically significant (p=0.0022). Thus, this study indicates that newborns with a CRIB II score of more than 7 have a greater risk of mortality than neonates with a CRIB II score of less than 7.

**Table 6:** Association of CRIB II score with deaths CRIB II: Clinical Risk Index for Babies II; HS: highly significant

CRIB II score	Deaths	Survivors	Odds ratio (95% confidence interval)	P-value
>7	18 (69.23%)	4 (22.22%)	7.87 (1.67-41.64)	0.0022, HS
≤7	8 (30.77%)	14 (77.78%)		
Total	26	18		

## Discussion

A descriptive-analytical study encompassing the period from 2016 to 2017 was undertaken at a hospital in Tehran, Iran, focusing on 344 preterm neonates weighing below 1.5 kg at birth and <32 weeks by gestational age. Among this cohort, a total of 253 infants demonstrated survival within the initial 24 hours of life, whereas 91 succumbed to mortality. However, our study had a relatively small sample size of 44, of which 18 newborns survived (40.91%) and 26 babies died (59.09%). In our investigation, the estimate of the ROC is 0.83 with a cut-off point of 7, whereas in the other study, the ROC was assessed to be 0.84 with a cut-off point of 8.5. In this cross-sectional study conducted in Tehran, the positive predictive value, negative predictive value, sensitivity, and specificity of the CRIB II score were 55%, 89.5%, 75%, and 78%, respectively. A significant association was seen between mortality and CRIB II score [10].

Researchers from the Korean Neonatal Network (KNN) conducted a study on a large national cohort to test the CRIB II score in the prediction of mortality according to the time after birth and also to predict the short-term morbidities of very-low-birth-weight (VLBW) newborn [11]. In accordance with their findings, the strength of CRIB II for the prediction of mortality surpassed that of relying solely on birth weight or gestational age as individual parameters. Henceforth, CRIB II demonstrated notable performance within the initial 30 days (AUC-0.8435) and from days 31 to 90 (AUC-0.8458) while exhibiting diminished efficacy beyond the 90-day threshold (AUC-0.6576).

A prospective observational study was performed at a tertiary care institute in central India, focusing on 140 neonates with a gestational age range from 28 to 31 weeks and birth weight within the range of 1000-2499 grams, who were admitted to the NICU. The included newborns exhibited a mean gestational age of 30.27±0.89 weeks and a mean birth weight of 1599.75±282.35 grams. The mean CRIB II score among survived newborns was 5.66±2.24, and among newborns who died, it was 13.16±25.56 with a range of 1-19. This study showed that as the CRIB II score increased, mortality increased progressively while the percentage of survival increased with increasing gestational age, hospital stay, birth weight, and body temperature (for each variable p<0.0001), but in our study, there was no significant association between the survival of newborns and individual factors such as gestational age, age in hours at hospital admission, birth weight of the baby, or sex of the baby. However, an increase in the period of hospital stay was associated with lower odds of survival. The AUC was 0.9868 for the ROC curve for the CRIB II score, while in our study, it was 0.83. This study concluded that the CRIB II score is of use for deciding on the needed interventions in the NICU and can help in the reduction of mortality in neonates [12].

The main goal of a second Indian study, which involved 44 preterm infants, was to determine whether there is a correlation between the SNAP II score and CRIB II score. The area under the ROC curve was used to express the prediction accuracy of birth weight, gestational age, the SNAP II score, and the CRIB II score. The CRIB II score (AUC 0.909) demonstrated stronger discrimination than the SNAP II score (0.869). Similar to our study, ROC analysis showed that birth weight and gestational age were not good in the prediction of death when compared to CRIB II. CRIB II (AUC=0.556) demonstrated stronger discrimination than SNAP-II (AUC=0.404) when the two scores were compared to see which one better predicts the overall newborn morbidity, demonstrating that CRIB II was superior to SNAP-PE II overall [13].

Eighty low-birth-weight newborns were examined in a study by Stomnaroska and Danilovski. Maternal co-morbidities and gestational age were substantially linked with neonatal mortality, but not with birth weight. The lowest value of the Apgar score had a positive correlation with gestational age, but didn’t have any correlation with the birth weight. This indicates that birth weight and gestational age are better in predicting mortality (AUC 0.923 and 0.904) than CRIB II (AUC 0.861) [14]. Nevertheless, our research revealed that the mortality predictive values of birth weight and gestational age were low.

In West Bengal, 143 VLBW babies were admitted to the North Bengal Medical College & Hospital's NICU in Darjeeling, as part of a prospective cohort study. Following their discharge, the babies were monitored. With a mean of 1199.6 and a median of 1240 grams, the range of birth weight was from 500 to 1500 grams. The CRIB II score has a mean of 8.021 and a range of 1-18 as compared to our study which showed a median CRIB II score among survivors of 6 with a range of 1-12, while among those who expired, it was 9.5 with a range of 5-14. CRIB II score was determined to be very useful for VLBW neonates in terms of mortality and neuro-developmental assessment [15].

A meta-analysis and systematic review of birth weight, gestational age, and six neonatal disease severity scores (CRIB, CRIB II, SNAP, SNAP II, SNAP-PE, and SNAP-PE II) was conducted by the authors using data sources published up to January 2019 from PubMed, Scopus, and EMBASE websites. It should be noted that only 24 of the 1,659 studies met the requirements for inclusion [16]. While gestational age was the least discriminating predictor of mortality (AUC 0.76), the CRIB index was the most accurate (AUC 0.88) [16].

The relatively small sample size and the potential for selection bias are the main limitations of the study. The study may not account for all possible confounders that could influence the outcomes being studied. Also, since the study was conducted in a single center, its findings may not be applicable to other settings with different patient populations or resources.

## Conclusions

This study suggests that neonates with a CRIB II score greater than 7 have a higher mortality risk than the neonates with a CRIB II score less than 7. Also, this study suggests that with increasing CRIB II score, there is an increased risk of mortality in newborns. The CRIB II score is a valuable tool for predicting mortality in newborns. However, further research with larger sample sizes or multicenter studies, which are more representative, are needed to expand upon these findings.
